# Research on the influence of the visual perception characteristics of fitness trail landscape space based on psychological perception: a case study of Hunnan District, Shenyang, China

**DOI:** 10.3389/fpsyg.2025.1595451

**Published:** 2025-05-01

**Authors:** Wenli Zhu, Zikun Chen, Xiaojun Wang, Feng Hu

**Affiliations:** School of Architecture and Urban Planning, Shenyang Jianzhu University, Shenyang, China

**Keywords:** fitness trail, visual perception of landscape space, psychological perception, image semantic segmentation, SD method

## Abstract

Based on the multidisciplinary method of collaborative psychology, sociology and environmental design research, this study conducts a quantitative study on the relationship between the visual perception of fitness trail landscape space and the psychological perceptions of fitness activities, providing data-driven references for the design of fitness trials. First, self-image acquisition is used to form a fitness trail landscape space image material library including 646 pictures. Second, we identify the user preferences for the composition of the main landscape elements of fitness trails, with subsequent research focusing on the top two preference combinations, namely, “plant–plant” and “plant–water.” Third, on the basis of the results of the semistructured interviews, a word frequency analysis is conducted, and the visual perception feature index framework of the fitness trail landscape space containing 12 indicators is constructed in combination with the relevant literature. An image semantic segmentation process, along with manual auditing, is applied to the self-collected images, resulting in a dataset of visual perception characteristic indicators. Furthermore, based on the KANO model, a psychological perception evaluation framework is constructed, and psychological perception evaluation datasets are obtained by using the semantic differential (SD) method. Finally, correlation analysis, multiple regression, and curve fitting are applied to the two datasets and the relevant threshold ranges are obtained. The results show that within the “plant–plant” combination, the optimal threshold for the degree of enclosure is 66–81%, the optimal threshold for the green view rate is 62–78%, the optimal threshold for the degree of openness of the sky is 2.2–3.2%, and the optimal threshold for the building façade area ratio is 0–10%. For the “plant–water” combination, the optimal threshold for the green view rate is 58–68%, the optimal threshold for the openness of the sky is 4.1–6.0%, the optimal threshold for the water area ratio is 0.75–1.75%, and the optimal threshold for the road area ratio is 33–43%.

## 1 Introduction

Fitness trails are specifically designed urban infrastructures aimed at promoting physical activity and fostering a healthy lifestyle (Taryn et al., 2020). The establishment of fitness trails is closely linked to health policies, with governments worldwide increasingly emphasizing the improvement of public health infrastructure in cities as a response to the obesity crisis and related diseases (US Department of Health and Human Services, 2015; Wang et al., 2013). In the United States, fitness trails are defined as “walking and running routes designed with health objectives in mind, “typically located around parks, communities, and schools (Götschi and Loh, 2017). In Europe, many cities have integrated fitness trails into broader networks of walking and cycling paths, striving to create health-friendly urban environments (Kyle et al., 2023; Stefan et al., 2022). In China, according to the ‘Million Kilometer Fitness Trail Project Implementation Plan’ issued by the General Administration of Sport of China, fitness trails include walking trails, cycling trails, etc., which are organically combined with the construction of park green space and equipped with necessary supporting facilities such as identification systems, such as trail markers (Kai and Wang, 2020). Moreover, the definition of fitness trails has gradually evolved into a comprehensive health promotion space, which not only includes the function of physical fitness but also emphasizes the promotion of social and mental health. (Cong et al., 2021).

The American cognitive psychologist James Jerome Gibson proposed that, among the five basic human senses, visual perception is the most important for spatial environment information stimulation (Niu and Li, 2022; Liu et al., 2023). The influence of landscape spatial visual perception characteristics on psychological perceptions has become a significant topic in recent urban environment studies. Kaplan (1989) introduced the concept of “perceived quality, “emphasizing the key role of landscape spaces in shaping psychological perceptions. Gifford (2002) noted that well-designed landscapes can provide a sense of safety and comfort, encouraging individuals to engage with their surroundings. Hsieh and Lee (2010) elucidated the corresponding relationship between spatial elements and subjects’ psychological perception, demonstrating the impact of open spaces on the psychological sense of security in visual terms. Adam et al. (2012) proposed that green landscapes, as a fundamental visual characteristic, can have a positive effect on both physical and mental health. Research by Zhang and Li (2020) suggested that there is a profound connection between visual perception characteristics and emotional responses, indicating that good landscape design can optimize mental states and enhance overall well-being. Zhang et al. (2021) further confirmed the close relationship between visual perception characteristics and individual feelings of comfort and safety.

Based on the above, with the increasing demand of urban residents for fitness trails, future research and practice should explore the potential of fitness trail environment design for human psychological impact. In addition, the current related research is mainly based on clear influencing factors and lacks quantitative conclusions. Therefore, this study adopts a multidisciplinary approach that integrates psychology, sociology, and environmental design to explore a quantitative landscape spatial design model based on psychological perceptions, and to form a quantitative threshold on the basis of clarifying the influencing factors of visual perception of fitness trail landscape space, so as to support the design of sports environment. Subsequently, a mental health-oriented urban public health infrastructure development strategy can be formulated to provide an important basis for the formulation of urban health policies, so as to deal with urban health problems.

This study follows the logical path of collecting environmental information in the form of images, performs quantitative analysis between the visual perception caused by images and the psychological perceptions required for fitness activities (Supplementary Figure 1), and conducts in-depth research on the landscape space of fitness trails.

## 2 Methods

### 2.1 Study area

The study area is located in the Hunnan District of Shenyang City, Liaoning Province, China (Supplementary Figure 2). As of December 2024, the study area contains five fitness trails, including the Tandi Park Fitness Trail, Olympic Park Fitness Trail, Citizen Park Fitness Trail, Mozi Mountain Park Fitness Trail, and Neighborhood Park Fitness Trail, setting it as the research object. These trails differ in spatial structure, element composition, and facility type, which is beneficial for subsequent data extraction to form a comprehensive dataset.

### 2.2 Research methods and data

This study follows a five-step process (Supplementary Figure 3). First, self-image acquisition is used to form a fitness trail landscape space image material library. Second, based on survey results, the preference for the composition of the main landscape elements of the fitness trails is determined. Third, a word frequency analysis is conducted on the basis of the interview materials and combined with the relevant literature, and a framework for the visual perception characteristic indicators of fitness trail landscapes is constructed. Image semantic segmentation technology and manual auditing are employed to obtain the characteristic indicator dataset. Fourth, based on the KANO model, a psychological perception evaluation framework for fitness activities is developed, and a dataset of psychological perception evaluations is obtained using the semantic differential (SD) method. Finally, the relevant data are integrated, and a joint analysis is performed between the visual perception characteristic indicators of fitness trail landscapes and the psychological perception evaluation data of fitness activities.

#### 2.2.1 Image collection

For the five fitness trails studied, 273 sampling points (Group A) were selected at 50-meter intervals along the trails. Additionally, another 50 sampling points (Group B) were selected to increase the variability of visual perception characteristic indicators, resulting in a total of 323 sampling points (Table 1). Image collection occurred in September and October 2024, during clear weather conditions. Using a smartphone (model: ViVo X27, rear camera), images were captured from a human perspective at each sampling point along the forwards and backwards directions of the trail (Supplementary Figure 4). The camera height was set to 1.6 m, the elevation angle was set to 0°, and a focal length of 1x was used with an automatic white balance. In total, 646 images with a resolution of 1,080 × 1,440 pixels were obtained, forming the image material library.

**Table 1:** Fitness trail landscape space image acquisition point data.

Number	Fitness trail name	Length / km	Number of Group A	Number of Group B	Total
1	Tandi Park Fitness Trail	2.39	47	10	57
2	Olympic Park Fitness Trail	3.66	72	10	82
3	Citizen Park Fitness Trail	2.43	48	10	58
4	Mozi Mountain Park Fitness Trail	3.41	67	10	77
5	Neighborhood Park Fitness Trail	1.96	39	10	49
Total		13.85	273	50	323

#### 2.2.2 Study on preferences for the composition

The main landscape elements of the five fitness trails in the study area include plants, water bodies, terrain, and public service facilities. On the basis of these elements, five main built environment combinations were formed: “plant–plant,” “plant–water,” “terrain-water,” “plant-public service facilities,” and “plant-terrain.” Representative images for each combination were selected from the image material library, and a preference selection questionnaire supplemented with block model diagrams was developed (Table 2). The questionnaire was published on the Questionnaire Star website^1^ in the process of on-site investigation in October 2024.The respondents were individuals actively using the fitness trails, as well as nearby residents and workers who had previously used the trails, ensuring that they had a certain level of familiarity with the fitness trail space. All subsequent surveys followed this principle for distribution. A total of 87 valid questionnaires were collected. The gender distribution was 40.2% male and 59.8% female. The results (Table 2) revealed that the top two preferred combinations were “plant–water” (39.58%) and “plant–plant” (32.08%). Subsequent research will focus on these two combinations.

**Table 2:** Block model schematic and summary of the questionnaire results.

Number	Main built environment combinations	Pictures of the questionnaire		Results of the questionnaire	
		Realistic picture	Schematic diagram	Number of times selected	Proportion
1	Plant–plant	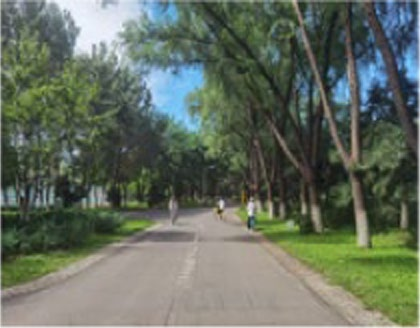	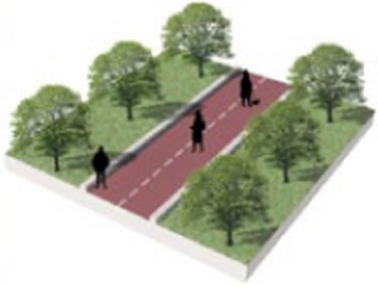	95	39.58%
2	Plant-water	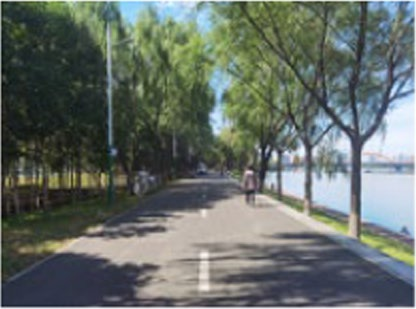	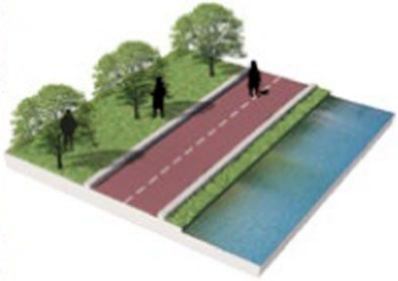	77	32.08%
3	Terrain-water	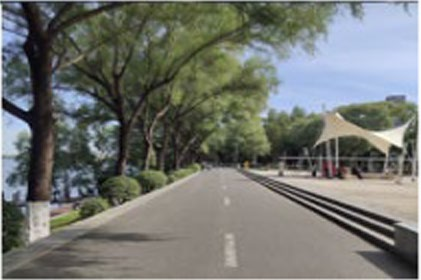	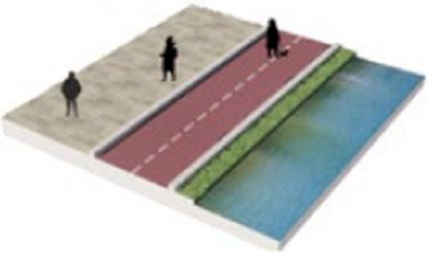	35	14.58%
4	Plant-public service facilities	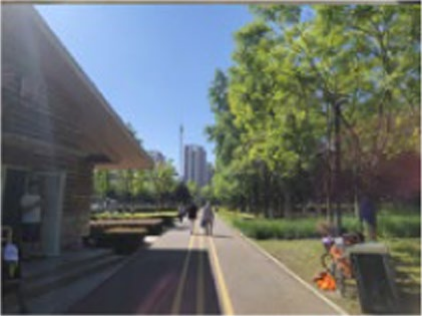	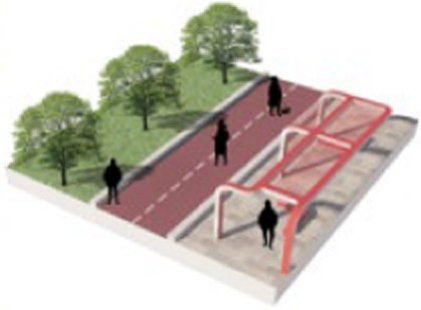	23	9.58%
5	Plant-terrain	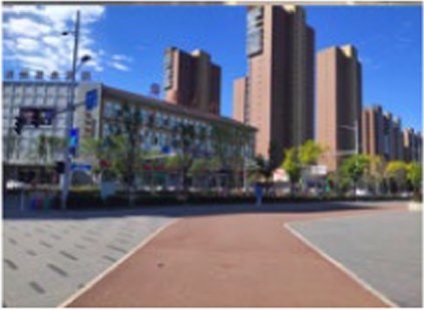	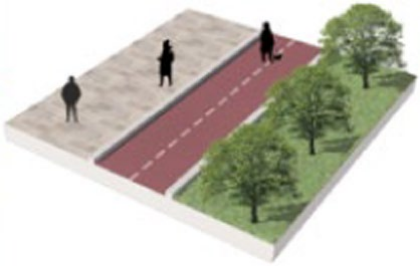	10	4.17%
Total				240	100.00%

#### 2.2.3 Framework construction and data processing of visual perception characteristic indicators

Semistructured interviews were conducted with 50 users of the fitness trials in the study area. The interview recordings were transcribed into text, yielding a total of 38,000 words. Word frequency analysis was performed using NVIVO software, with the minimum word length set to “2” and the “synonym” option selected, while other settings were kept at default. The results identified six effective interview categories: plants, color, roads, public service facilities and landscape richness. These categories were converted into quantifiable indicator elements. The relevant literature was also reviewed, leading to the identification of 12 characteristic indicators, which were grouped into 3 dimensions to form the final framework for fitness trail landscape spatial visual perception characteristic indicators (Table 3).

**Table 3:** Index framework and quantitative method of visual perception characteristics of fitness trail landscape space.

Characteristics dimension	Characteristic indicators	Definition and quantitative calculation	Quantitative way	Literature support
Surrounding environment	Degree of enclosure	The degree of being surrounded by the surrounding environment. The quantitative method is building or fence or green area / image area.	Image semantic segmentation	Medjkoune et al. (2023) Xingli et al. (2022) Baran et al. (2018) Butler and Kring (1987)
	Green-looking rate	The proportion of green plants in the range of human eyes. The quantization method is plant area / image area.		Jing et al. (2024) Liu et al. (2024) Zhu et al. (2024) Zhang and Chang (2022) Chen et al. (2020) Wang and Ze (2013)
	Openness of the sky	The proportion of the sky in the range of the human eye. The quantization method is sky area / image area.		Fu et al. (2025) Sun et al. (2024) Ren X et al. (2023) Ah (2016)
	Building facade area ratio	The proportion of building facades within the scope of the human eye. The quantization method is the building facade area / image area.		Othman (2017)
	Water area ratio	The proportion of water area in the range of human eyes. The quantization method is water area / image area.		Li (2024) Xingcan et al. (2023) Dong et al. (2021) Abdullah et al. (2020)
Facility configuration	Road area ratio	The proportion of roads within the scope of the human eye. The quantization method is the fitness trail area / image area.		Xingli et al. (2022) Bingqian et al. (2021)
	Public service facilities area ratio	The proportion of public service facilities within the range of human eyes. The quantification method is the sum of the area occupied by various public service facilities / image area.		Cao et al. (2024)
	The number of public service facility types	The number of categories of public service facilities identified in the segmented image.		Park (2014)
Integrated design	Landscape richness	The total number of landscape categories identified and segmented in the image.		Yan et al. (2014) Kalivoda et al. (2014)
	Color category richness	The color in the picture occupies the number of color series colors.	Manual measurement and audit	Ren Z et al. (2023) Wang et al. (2023) Fan et al. (2023)
	Plant class richness	The number of plant species in the image.		Yang et al. (2024) ANDÚJAR et al. (2010) Ing et al. (2010)

The quantification methods for the visual perception characteristic indicators of the fitness trail landscape space, which include both image semantic segmentation and manual auditing techniques, are outlined in Table 3. Image semantic segmentation was based on the GluonCV computer vision deep learning toolkit under the MXNet deep learning library (Jian et al., 2020), which includes two main components: model training and correction, and segmentation recognition. In the model training and correction phase, 25% of the images in the image dataset were annotated using the Labelme annotation software, generating corresponding JSON data files for the object features, thereby forming the foundational dataset. Subsequently, the ADE20K dataset and the foundational dataset were integrated to create a basic database, which was locally trained to correct the existing model and establish a suitable recognition model. Through cross-validation with multiple annotators, the model’s accuracy reached 90% of the results obtained from manual annotations, making it suitable for data extraction in this study. Segmentation recognition was then carried out based on this model. A total of 646 images from the image material library were input into this model to obtain the image recognition results (as shown in Supplementary Figure 5) and the area proportion data for common objects (if the data are 0, the object is not present in the image). The data were organized, and erroneous results were manually removed by comparison with the images. In the indicator data extraction process, the green view rate was represented by the sum of the data for “tree,” “grass,” and “plant,” and the road area was represented by the sum of the data for “road,” “sidewalk,” and “path.” Owing to the color category richness and plant class richness, which cannot be derived from image semantic segmentation, virtual manual auditing was employed to count their types. The auditing team consisted of four professionals with an architectural background, and after training, the consistency of the sample test results reached approximately 93%.

Thus, a dataset of visual perception characteristic indicators for the fitness trail landscape space was obtained based on the 646 self-collected images. To reduce the number of questionnaires used in subsequent research and improve the feasibility of the experiment, the following principles were applied for manual selection and data merging: (1) the images had similar weather, lighting, and sky color conditions; (2) the values of each characteristic indicator formed a certain level of difference and satisfied hierarchical changes. Ultimately, a total of 80 research images were selected (40 images for the “plant–plant” combination and 40 images for the “plant–water” combination) for subsequent psychological perception evaluation studies.

#### 2.2.4 Framework construction and data acquisition for psychological perception evaluation

On the basis of domestic and international literature, eight types of psychological perceptions related to landscape spatial visual perception are summarized: a sense of security, a sense of comfort, a sense of belonging, a sense of fun, a sense of pleasure, a sense of experience, a sense of brightness, and a sense of warmth. The KANO model was used to rank the importance of these psychological perceptions in fitness activities. Specifically, an online questionnaire was distributed via the Questionnaire Star website in November 2024. A total of 112 questionnaires were collected, and after excluding invalid questionnaires with consistent answers to positive or reverse questions, 109 valid responses were obtained. The data generated by the questionnaire showed high reliability and validity, with Cronbach’s *α* value of 0.892 and KMO value of 0.884. Additionally, research indicates that the improved better-worse coefficient method based on the traditional KANO model proposed by Berger et al. can enhance the accuracy of demand attribute classification (Yi et al., 2024). This study applied this method for quantitative analysis and classification, creating a quadrant chart (Supplementary Figure 6) and obtaining attribute classification results (Table 4). The results showed that the sense of security is a one-dimensional quality, whereas the sense of pleasure and the sense of experience are attractive qualities. The remaining psychological perceptions were categorized as indifferent qualities. Therefore, the psychological perception evaluation framework for fitness activities was established on the basis of a sense of security, a sense of pleasure, and a sense of experience.

**Table 4:** KANO model attribute classification analysis results.

Psychological perceptions	M (Must-be quality)/%	O (One-dimensional quality)/%	A (Attractive qualities)/%	I (Indifferent qualities)/%	R (Reverse quality)/%	Q (Questionable result)/%	Attribute	Better	Worse
Sense of security	19.61	30.39	13.73	27.45	0	8.82	M	48.39	−54.84
Sense of pleasure	3.92	14.71	44.12	21.57	1.96	13.73	A	69.77	−22.09
Sense of experience	1.96	17.65	37.25	32.35	0	10.78	A	61.54	−21.98
Sense of fun	1.96	13.73	35.29	37.25	0	11.76	I	55.56	−17.78
Sense of comfort	2.94	18.63	25.49	40.2	1.96	10.78	I	50.56	−24.72
Sense of warmth	0.98	16.67	24.51	48.04	0.98	8.82	I	45.65	−19.57
Sense of belonging	1.96	11.76	26.47	49.02	0	10.78	I	42.86	−15.38
Sense of brightness	2.94	17.65	19.61	50.98	0.98	7.48	I	40.86	−22.58

A seven-point scale using the SD method was employed to form the questionnaire, and values were assigned to the seven levels for the three psychological perceptions (Table 5). Psychological perception evaluation data were collected for the 80 research images. An online questionnaire was distributed via the Questionnaire Star website in November 2024, resulting in 129 valid responses. The gender distribution was 39.2% male and 60.8% female. All the participants were ensured to have normal uncorrected or corrected-to-normal vision, with no color blindness or color vision impairments. Each participant completed 10 evaluations, ensuring that each image was evaluated at least 17 times. To familiarize the participants with the average image level, they were asked to browse 15 typical images before completing the questionnaire. The results were used to generate the psychological perception evaluation dataset for fitness activities. The data generated from the questionnaire demonstrated a high level of reliability, with Cronbach’s *α* values exceeding 0.9. Given the established structure of the questionnaire, validity testing was deemed unnecessary.

**Table 5:** The seven-point scale assignment results of the SD method.

Sense of security is extremely strong	7	6	5	4	3	2	1	Sense of security is extremely weak
Sense of pleasure is extremely strong	7	6	5	4	3	2	1	sense of pleasure is extremely weak
Sense of experience is extremely strong	7	6	5	4	3	2	1	sense of experience is extremely weak

#### 2.2.5 Data joint analysis calculus

IBM SPSS Statistics 20 software was used to analyze the fitness trail landscape spatial visual perception characteristic indicator data (independent variables) and the psychological perception evaluation data (dependent variables; Zheng et al., 2023). First, Pearson correlation analysis was performed on the continuous variables (landscape spatial visual perception characteristic indicators and psychological perception evaluation data) to obtain the correlation coefficients and corresponding *p* values (Ren et al., 2024). Significant correlations (*p* < 0.05) were identified. Next, multivariate regression analysis was conducted on the significant dependent and independent variables, and the degree of influence was determined based on the standardized coefficient beta values. Scatter plots were then created with the independent variables on the x-axis and the dependent variables on the y-axis. Various mathematical models were used to fit the curves, and the model with the best fit was selected to create an overall trend graph. Finally, on the basis of the trend graph, the range of characteristic indicators at which the three psychological perceptions reach their peak values was identified to provide design-related data references.

## 3 Research results and statistical analysis

### 3.1 Correlation analysis

Table 6 presents the results of the correlation analysis between the visual perception feature indicators of the fitness trail landscape and the psychological evaluation data for the two combinations. On the basis of the correlation coefficients and *p* values, in the “plant–plant” combination, the degree of enclosure and the green-looking rate were significantly positively correlated with the three types of psychological perceptions. The openness of the sky is significantly positively correlated with the sense of experience and negatively correlated with the sense of safety and pleasure. The building facade area ratio is significantly negatively correlated with pleasure and experience. The number of public service facility types and landscape type richness Landscape richness are both significantly negatively correlated with pleasure. The other factors were not significantly correlated. In the “plant–water” combination, the green-looking rate was significantly positively correlated with the sense of safety and pleasure. The openness of the sky is significantly negatively correlated with a sense of safety and pleasure. The water area ratio and Landscape richness are significantly negatively correlated with the sense of safety, and the road area ratio is significantly positively correlated with the sense of safety. The other factors were not significantly correlated. Furthermore, when comparing supporting facilities and overall design, the correlation within the characteristic dimension of the surrounding environment was found to be more closely related.

**Table 6:** Correlation results between the landscape space visual perception characteristic index of the fitness trail and the psychological perception evaluation.

Characteristics dimension	Characteristic indicators	Types value	Plant–plant			Plant-water		
			Sense of security	Sense of pleasure	Sense of experience	Sense of security	Sense of pleasure	Sense of experience
Surrounding environment	Degree of enclosure	Correlation coefficient	0.319*	0.520**	0.422**			
		*p*	0.045	0.001	0.007			
	Green-looking rate	Correlation coefficient	0.352*	0.527**	0.486**	0.376*	0.431**	
		*p*	0.026	0.000	0.001	0.017	0.005	
	Openness of the sky	Correlation coefficient	−0.388*	−0.576**	0.483**	−0.436**	−0.488**	
		*p*	0.013	0.000	0.002	0.005	0.001	
	Building facade area ratio	Correlation coefficient		−0.375*	−0.443**			
		*p*		0.017	0.004			
	Water area ratio	Correlation coefficient				−0.353*		
		*p*				0.026		
Facility configuration	Road area ratio	Correlation coefficient				0.319*		
		*p*				0.045		
	Public service facilities area ratio	Correlation coefficient						
		*p*						
	The number of public service facility types	Correlation coefficient		−0.352*				
		*p*		0.026				
Integrated design	Landscape richness	Correlation coefficient		−0.402*		−0.365*		
		*p*		0.010		0.021		
	Color category richness	Correlation coefficient						
		*p*						
	Plant class richness	Correlation coefficient						
		*p*						

* indicates that *p* < 0.05 indicates that the correlation is significant; ** indicates that *p* < 0.01 indicates that the correlation is extremely significant.

### 3.2 Analysis of the influence

A multiple regression analysis was conducted on the significantly correlated independent and dependent variables. To eliminate differences in the units of independent variables, standardized coefficients (beta) were used to represent the extent of influence of various fitness trail landscape spatial visual perception characteristics on psychological perception evaluation. Furthermore, to avoid the impact of multicollinearity on the effectiveness of beta coefficients, this study performs secondary standardization for independent variables with a variance inflation factor (VIF) greater than 10 and absolute beta values greater than 1. The results and beta values are shown in Table 7.

**Table 7:** Multiple regression analysis results of the landscape space visual perception characteristic index of fitness trail and psychological perception evaluation.

Characteristics dimension	Characteristic indicators	Standardized coefficients (Beta)				
		Plant–plant			Plant-water	
		Sense of security	Sense of pleasure	Sense of experience	Sense of security	Sense of pleasure
Surrounding environment	Degree of enclosure	−0.367	0.058	0.043		
	Green-looking rate	0.365	0.170	0.525	0.791	0.087
	Openness of the sky	−0.411	−0.477	−0.500	0.328	−0.416
	Building facade area ratio		−0.196	−0.413		
	Water area ratio				−0.177	
Facility configuration	Road area ratio				0.539	
	The number of public service facility types		−0.051			
Integrated design	Landscape richness		−0.041		−0.200	

From Table 6, in the “Plant–Plant” combination, the influence of the independent variables on the sense of safety, ranked from highest to lowest, is as follows: the degree of openness of the sky, the degree of enclosure, and the green-looking rate. For the sense of pleasure, the influence from highest to lowest is as follows: the degree of openness of the sky, the building façade area ratio, the green-looking rate, the degree of enclosure, the number of public service facility types, and landscape richness. For the sense of experience, the influence from highest to lowest is the green-looking rate, the openness of the sky, the building façade area ratio, and the degree of enclosure. For the “plant–water” combination, the influence of the independent variables on the sense of safety, ranked from highest to lowest, is as follows: the green-looking rate, road area ratio, openness of the sky, landscape richness, and water area ratio. For the sense of pleasure, the influence from highest to lowest is the openness of the sky and the green-looking rate.

### 3.3 Curve fitting analysis

A comparison of various mathematical models revealed that the cubic model presented the highest degree of fit. Therefore, the cubic model fitting results were used in this study (Table 8). Supplementary Figures 7–15 show the curve fitting images, with Supplementary Figures 7–12 showing the fitting results for the “Plant–Plant” combination and Supplementary Figures 13–15 showing the fitting results for the “Plant–Water” combination.

**Table 8:** Curve fitting results of the landscape space visual perception characteristic index of fitness trail and psychological perception evaluation.

The degree of curve fitting(R^2^)		Plant–plant			Plant-water	
Characteristics dimension	Characteristic indicators	Sense of security	Sense of pleasure	Sense of experience	Sense of security	Sense of pleasure
Surrounding environment	Degree of enclosure	0.237	0.330	0.279		
	Green-looking rate	0.244	0.323	0.322	0.173	0.231
	Openness of the sky	0.165	0.339	0.236	0.166	
	Building facade area ratio		0.184	0.226		
	Water area ratio				0.212	0.264
Facility configuration	Road area ratio				0.109	
	The number of public Service facility types		0.134			
Integrated design	Landscape richness		0.170		0.146	

For images with complete cubic curve characteristics, the interval corresponding to the peak of each curve is defined as the threshold range by taking the x-coordinate value of the peak ±5%. These threshold ranges are merged within each graph, and the final peak segment corresponds to the optimal threshold range for the feature indicators. For other images, the best threshold is determined by the inflection point.

Thus, in the “Plant–Plant” combination, according to Supplementary Figures 7, 8, with increasing degree of enclosure and green-looking rate, the curve fitting results for all the psychological evaluations show a “decline-rise-decline” trend. Since the y-axis values are overall greater than 4.00, the evaluations are positive. The peak segment occurs when the enclosure percentage is between 66 and 81% and when the green-looking percentage is between 62 and 78%. According to Supplementary Figure 9, openness of the sky shows a monotonically decreasing curve fitting result with all the psychological evaluations, indicating that greater openness of the sky leads to poorer psychological perceptions. The peak occurs when the openness of the sky is between 2.2 and 3.2%. According to Supplementary Figure 10, the building façade area ratio shows a monotonically decreasing curve fitting result with pleasure and experience, indicating that a larger facade area results in lower pleasure and experience. The psychological evaluation is positive when the building façade area ratio is less than 10%, with a smoother decreasing trend between 10 and 35% and a more pronounced decline beyond 35%, where the y-axis values fall below 4.00, indicating a negative evaluation. According to Supplementary Figures 11, 12, the number of public service facility types and landscape richness both show monotonically decreasing curve fitting results with pleasure, suggesting that higher values of these characteristics lead to lower pleasure. Since the y-axis values are overall greater than 4.00, the evaluations are positive. Additionally, Supplementary Figure 12 shows that the peak segment of the number of public service facility types and landscape richness lacks statistical significance.

In the “Plant–Water” combination, according to Supplementary Figure 13, the green-looking rate shows a “decline-rise-decline” trend with both a sense of safety and pleasure. Since the y-axis values are overall greater than 4.00, the evaluations are positive. The peak occurs when the green-looking rate is between 58 and 68%. According to Supplementary Figure 14, the degree of openness of the sky shows a “brief rise followed by a decline” trend with both a sense of safety and pleasure, with the peak occurring between 4.1 and 6.0%. According to Supplementary Figures 14, 15, the water area ratio, road area ratio, and landscape richness show different trends in their curves with respect to safety: “brief rise followed by a decline then rise,” monotonically increasing, and monotonically decreasing, respectively. Since the y-axis values are all greater than 4.00 in these graphs, the evaluations are positive. The peak water area ranges from 0.75–1.75%, and the peak road area ratio ranges from 33 to 43%. Additionally, Supplementary Figure 15 shows that the peak segment for landscape richness lacked statistical significance.

## 4 Conclusion and discussion

### 4.1 Study of the correlation and influence degree

This study focuses on the two combinations of “plant–plant” and “plant–water” and summarizes the correlation and degree of influence between fitness trail landscape spatial visual perception characteristics and psychological perception experiences (Table 9).

**Table 9:** Relationship between visual perception characteristics of fitness trail landscape space and psychological perception of fitness activities.

Combination mode	Psychological perception	Correlation	Characteristics dimension	Ranking of impact(the absolute value of beta)
Plant–plant	Sense of security	Positive	Degree of enclosure Green-looking rate	Openness of the sky(0.411)>degree of enclosure(0.367)>green-looking rate(0.365)
		Negative	Openness of the sky	
	Sense of pleasure	Positive	Degree of enclosure Green-looking rate	Openness of the sky(0.477)>Building facade area ratio(0.196)>green-looking rate(0.170)>degree of enclosure(0.058)>the number of public service facility types(0.051)>Landscape richness(0.041)
		Negative	Openness of the sky Building facade area ratio The number of public service facility types Landscape richness	
	Sense of experience	Positive	Degree of enclosure Green-looking rate Openness of the sky	green-looking rate(0.525)>Openness of the sky(0.500)>Building facade area ratio(0.413)>degree of enclosure(0.043)
		Negative	Building facade area ratio	
Plant-water	Sense of security	Positive	Green-looking rate Road area ratio	green-looking rate(0.791)>road area ratio(0.539)>Openness of the sky(0.328)>Landscape richness(0.200)>Water area ratio(0.177)
		Negative	Openness of the sky Water area ratio Landscape richness	
	Sense of pleasure	Positive	Green-looking rate	Openness of the sky(0.416)>green-looking rate(0.087)
		Negative	Openness of the sky	

With respect to the sense of safety, in the “Plant–Plant” combination, the independent variables influencing safety, ranked by degree of influence, are as follows: openness of the sky, degree of enclosure, and green-looking rate. This suggests that spatial openness plays a crucial role in enhancing individuals’ sense of safety in this combination. For the “Plant–Water” combination, the green-looking rate has a more significant impact, followed by the road area ratio and the openness of the sky. This difference may arise from the similar visual characteristics of water and the sky as visual elements (Sztuka et al., 2022), both of which promote psychological relaxation and comfort through their openness and natural elements. These can serve as interchangeable elements in urban spaces to improve psychological well-being (Sun et al., 2024).

With respect to the sense of pleasure, the factors influencing the “plant–plant” combination are more diverse. Compared with safety, additional indicators include the building façade area ratio and the number of public service facility types, with the building façade area ratio exerting a greater degree of influence, highlighting the importance of considering the harmony between architectural and natural elements (Zhang et al., 2025). In the “plant–water” combination, pleasure is influenced mainly by the degree of openness of the sky and the green-looking rate, indicating that the combination of water and open views most effectively enhances public enjoyment. This finding aligns with the research of Chen et al. (2022), who concluded that open spaces combined with water characteristics significantly promote physical and mental health.

With respect to the sense of experience, in the “Plant–Plant” combination, the influences of the green-looking rate, the openness of the sky, the building façade area ratio, and the degree of enclosure should not be overlooked. Although the degree of influence varies, the standardized beta coefficients are relatively close, indicating that designers should give attention not only to plant greening but also to the visual composition of buildings and their impact on spatial experience. This finding resonates with the conclusions of Ren Z et al. (2023), who emphasized the comprehensive impact of multidimensional factors on environmental experience.

In summary, the conclusions of this part align well with existing relevant literature, while also extending current research in terms of the influence degree. Although previous studies have explored the correlation between landscape features and psychological perception, none quantitative analysis or ranking of influence degree have conducted on the extent to which individual landscape features affect psychological experiences based on data results. This study conducts depth discussions on this blank area, providing clear priority of landscape features for fitness trail landscape design.

### 4.2 Threshold range study

The optimal threshold ranges for various landscape spatial visual perception feature indicators in fitness trails for the two combinations are summarized in Table 10. The results of this study can provide more precise empirical evidence for the design of fitness trails.

**Table 10:** The optimal threshold result of the visual perception feature index.

Number	Characteristic indicators	Plant–plant	Plant-water
1	Degree of enclosure	66–81%	—
2	Green-looking rate	62–78%	58–68%
3	Openness of the sky	2.2–3.2%	4.1–6.0%
4	Building facade area ratio	0–10%	—
5	Water area ratio	—	0.75–1.75%
6	Road area ratio	—	33–43%

With respect to the degree of enclosure, previous studies (Baran et al., 2018; Tabrizian et al., 2018) have noted that moderate enclosure helps enhance environmental comfort. It is necessary to avoid over-opening and avoid the formation of closed space. Our findings support this view, and at the same time, data analysis of this study shows that the optimal psychological perception is achieved when the degree of enclosure of the fitness trail is between 66 and 81%.

Jing et al. (2024) proposed a positive correlation between a high green-looking rate and psychological relaxation, whereas Liu et al. (2024) suggested that an excessively high green-looking rate might lead to an overly monotonous environment, which could suppress physical activity. Our results are consistent with the above conclusions, and at the same time, data analysis of this research shows that a high green-looking rate (over 78%) inhibits positive psychological perception. The optimal threshold ranges for the “plant–plant” and “plant–water” combinations are 62–78% and 58–68%, respectively.

Regarding the openness of the sky, Li and Du (2024) noted that moderate openness of the sky provides sufficient natural light and spatial perception, thereby promoting positive emotions and healthy behaviors. Additionally, studies conducted in different landscape contexts, such as American scholars Park and Ergan (2025) in street studies have also shown that excessive openness of the sky could increase feelings of exposure and anxiety, whereas too little openness could lead to a sense of confinement, decreasing the livability and attractiveness of the environment. Our findings are consistent with these studies. At the same time, through data analysis, this study finds that the optimal threshold ranges of “plant–plant” combinations and “plant-water” combinations are 2.2–3.2% and 4.1–6.0%, respectively.

With respect to the water area ratio, Dong et al. (2021) also pointed out that water can significantly improve emotions and comfort in the study of waterfront linear parks. Dinu Roman Szabo et al. (2023) found that water elements can effectively alleviate stress and fatigue, especially in public spaces such as trails. Our findings support this finding. At the same time, this study quantifies the water area ratio index, and obtains that it can maximize the psychological perception when it accounts for 0.75–1.75% in the visual range, which provides a quantitative basis for previous research.

In addition, this study also obtains the corresponding threshold results of building facade area ratio and road area ratio. These two landscape features have not been adequately emphasized in the existing literature, potentially contributing to differences in some research outcomes.

In summary, this study verifies the effects of the degree of enclosure, green-looking rate, the openness of the sky and water area ratio on psychological perception, and forms a good echo with the existing research. In addition, through quantitative analysis, the threshold range of the above six indicators including building facade area ratio and road area ratio are obtained. The results of this study fill the gaps in the lack of accurate threshold data for the fitness trail landscape feature indicators and provide a strong empirical basis for the design, which form an effective research methodology.

### 4.3 Research limitations and future prospects

This study does not account for the impact of day–night and seasonal changes in fitness trail landscape spatial characteristics on psychological perception. Considering the sunset times and climatic characteristics of Shenyang, Liaoning Province, future research could expand to investigate these aspects. Furthermore, regarding psychological perception evaluation, the subjectivity in respondents’ choice of options during questionnaire completion may raise concerns about the objectivity of the evaluation data. Future studies should aim to use validated scales to address this concern.

## Data Availability

The raw data supporting the conclusions of this article will be made available by the authors, without undue reservation.

## Footnotes

^1^ https://www.wjx.cn/
