# Supplementary material for: Research on the influence of the visual perception characteristics of fitness trail landscape space based on psychological perception: a case study of Hunnan District, Shenyang, China
Source: Front Psychol. 2025 May 1;16:1595451. doi: 10.3389/fpsyg.2025.1595451 (PMC12078341; doi:10.3389/fpsyg.2025.1595451)
Supplement: Supplementary file 1 [file Table_1.docx]

Supplementary Material

# Supplementary Figures and Tables

## Supplementary Figures

##
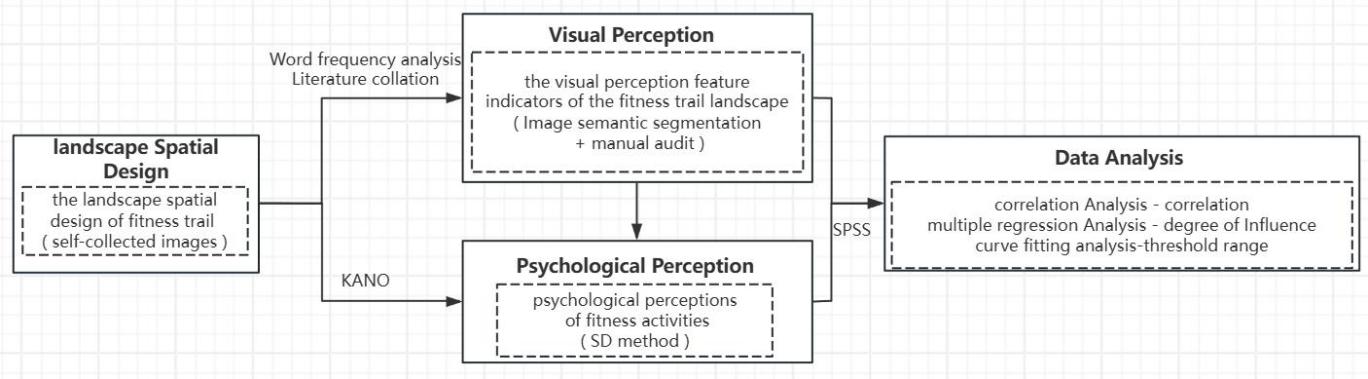


**Supplementary Figure 1.**Research on logical path graphs


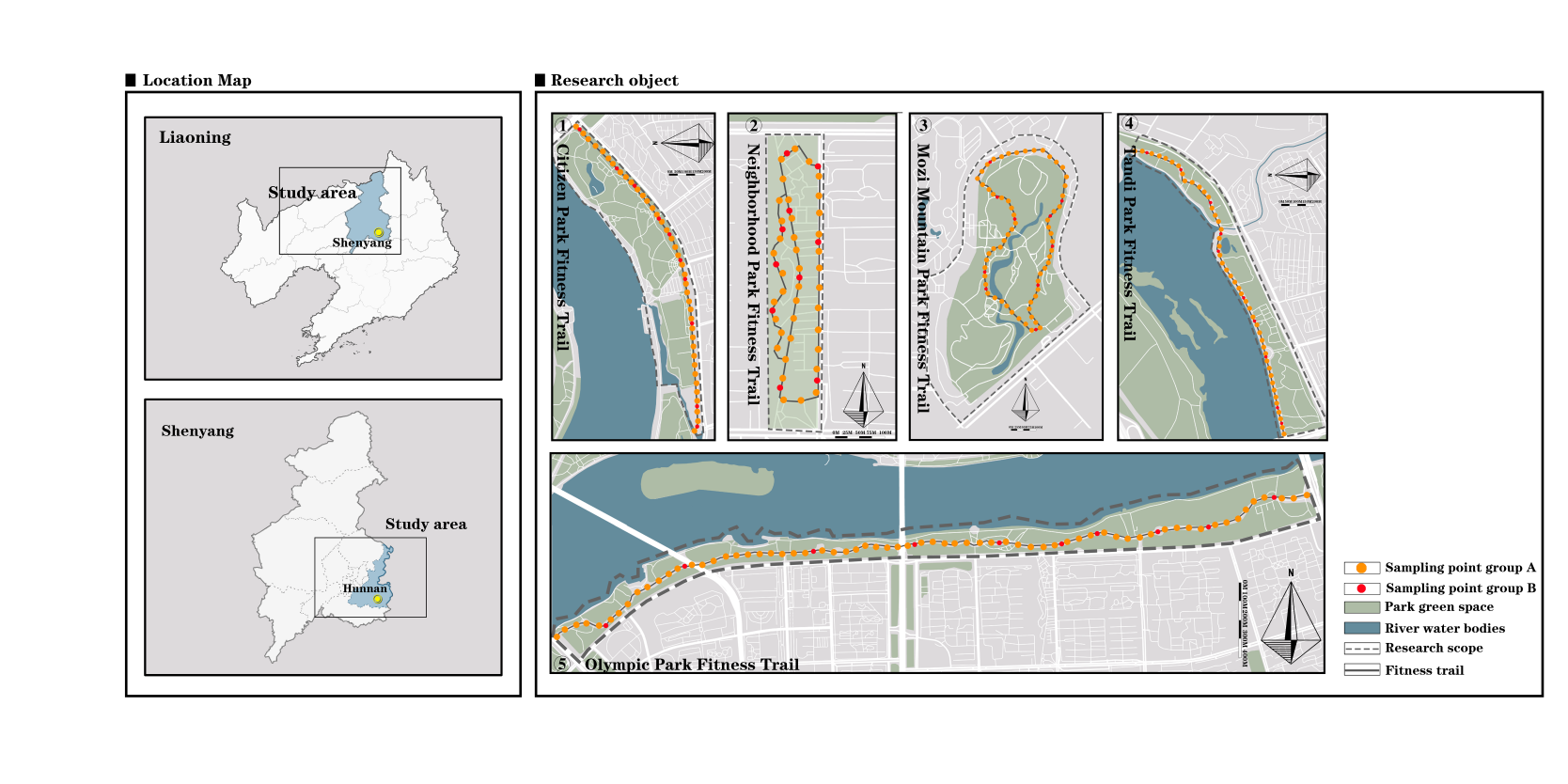


**Supplementary Figure 2.**Research area location and fitness trail distribution map


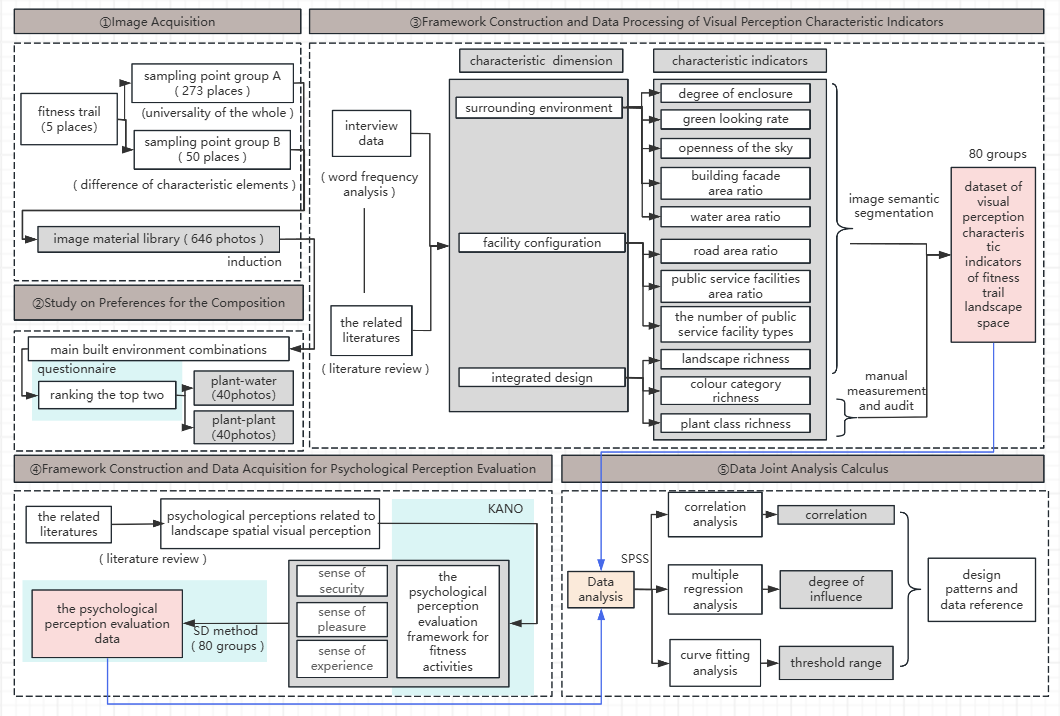


**Supplementary Figure 3.**Research flow chart


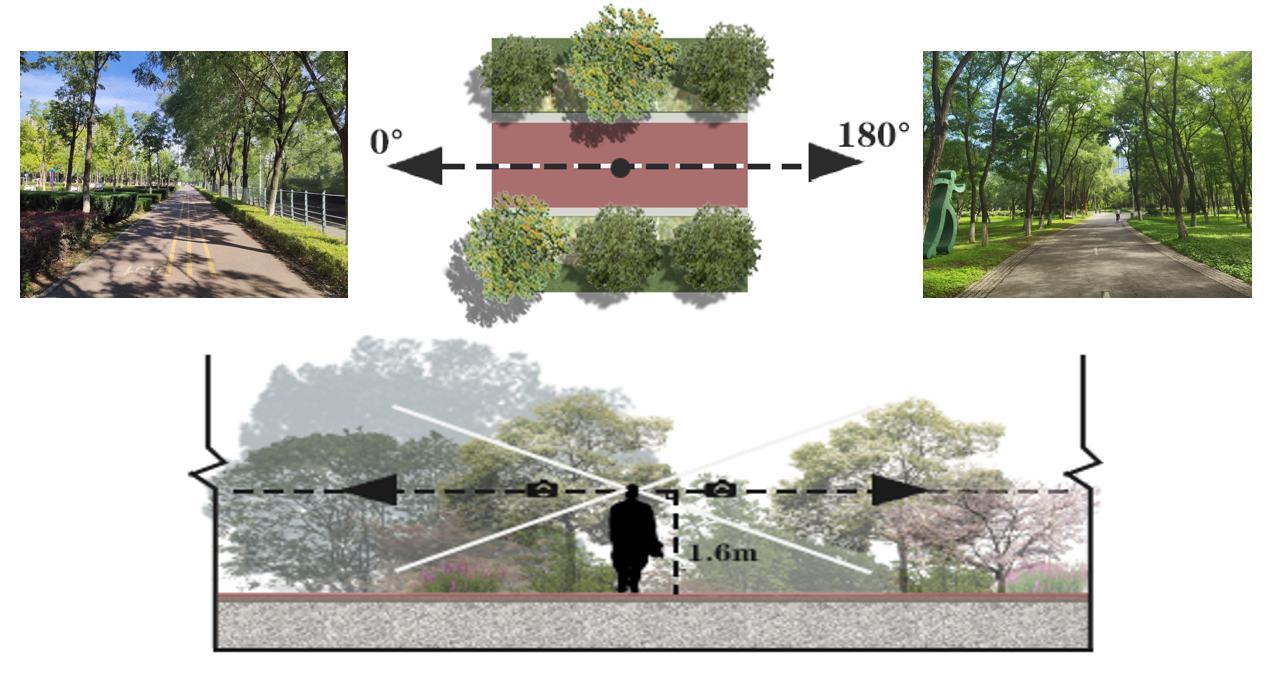


**Supplementary Figure 4.** Image acquisition schematic


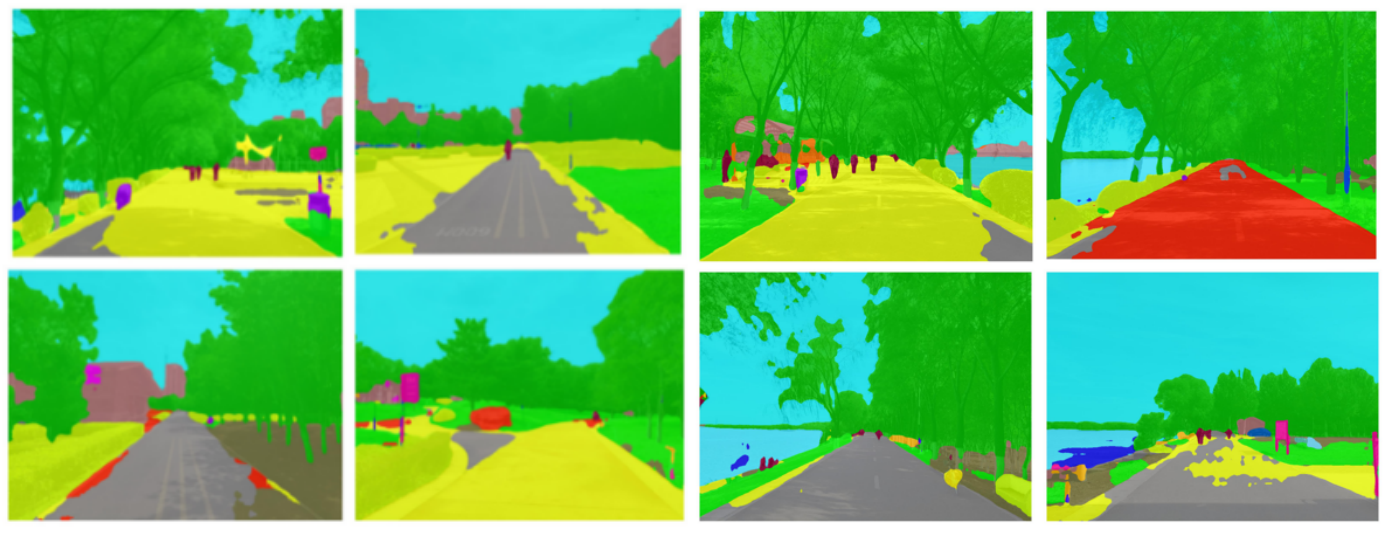


**Supplementary Figure 5**.Image semantic segmentation results sketch


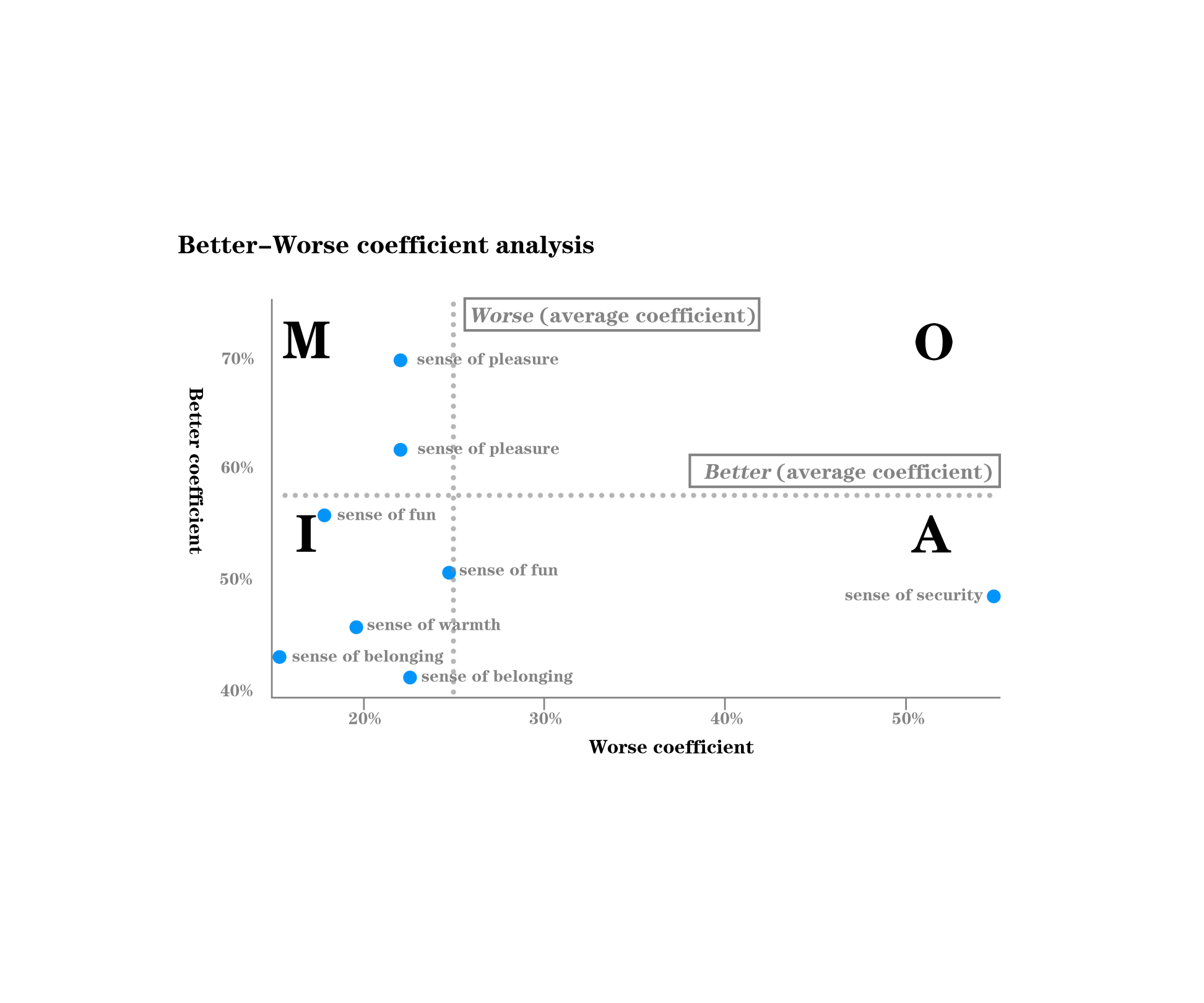


**Supplementary Figure 6.**Better-Worse coefficient quadrant diagram based on the KANO model


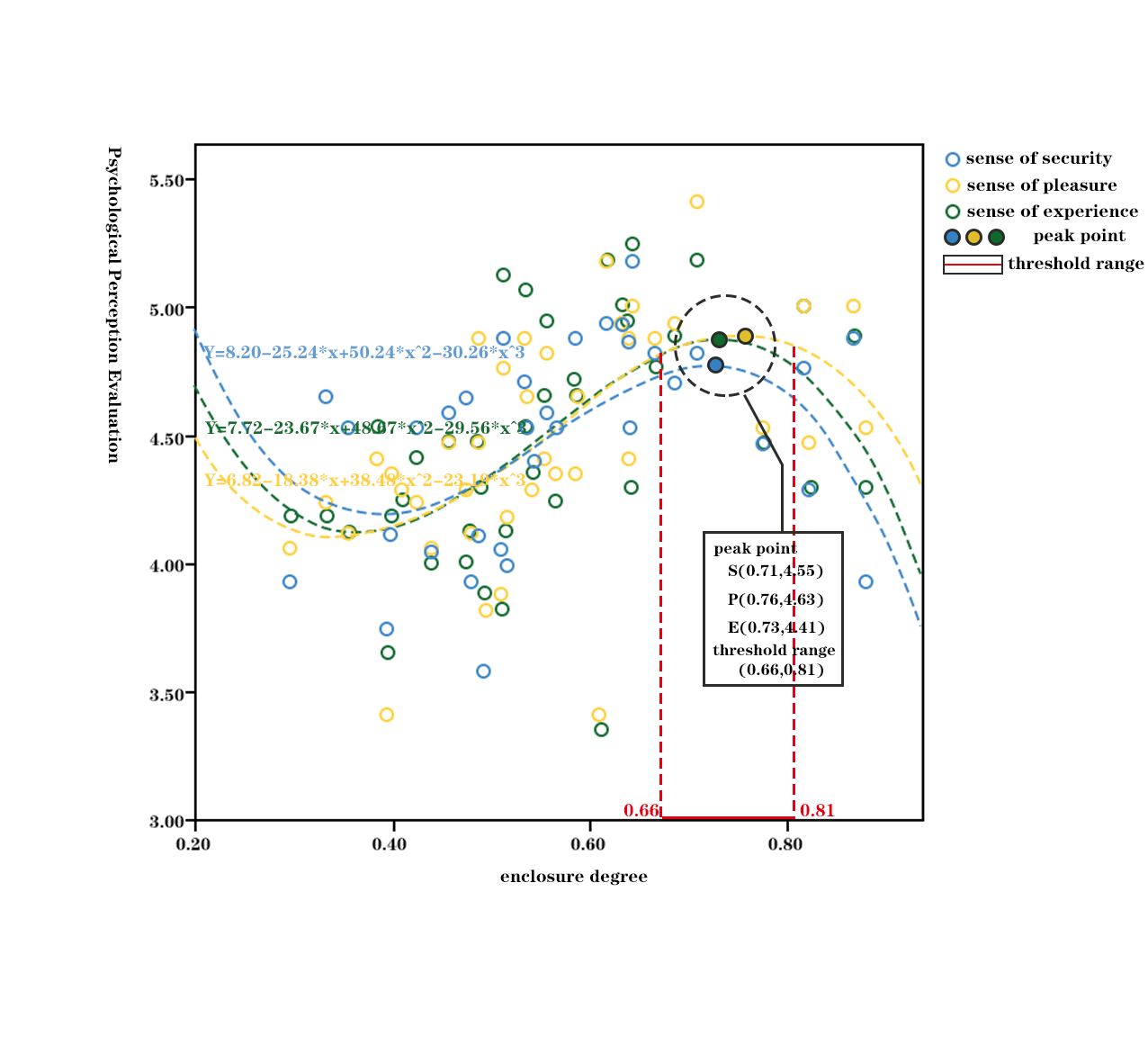


**Supplementary Figure 7.**Curve Fitting of the Degree of Enclosure and Three Psychological Perceptions


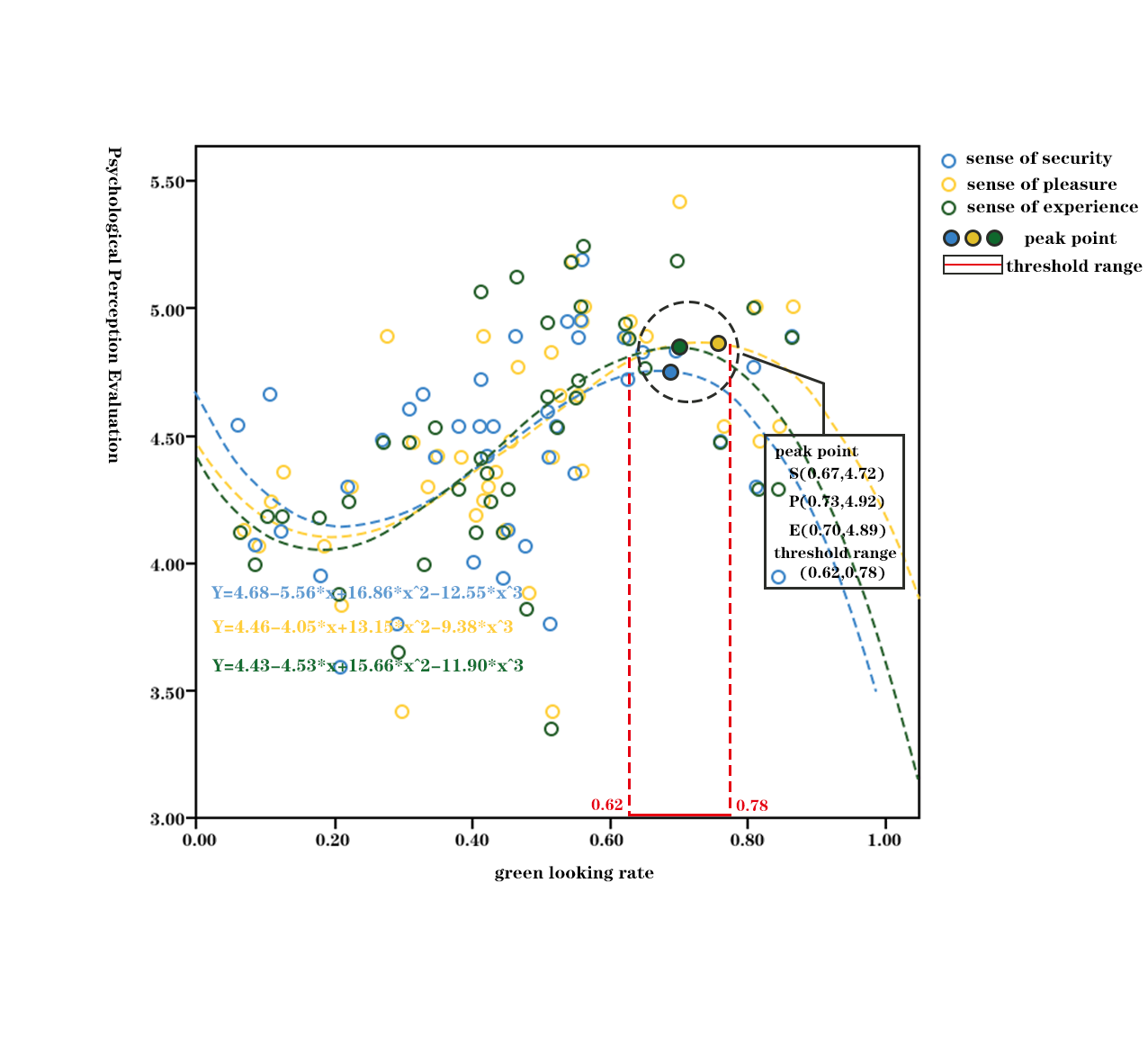


**Supplementary Figure 8.**Curve Fitting of the Green Looking Rate and Three Psychological Perceptions


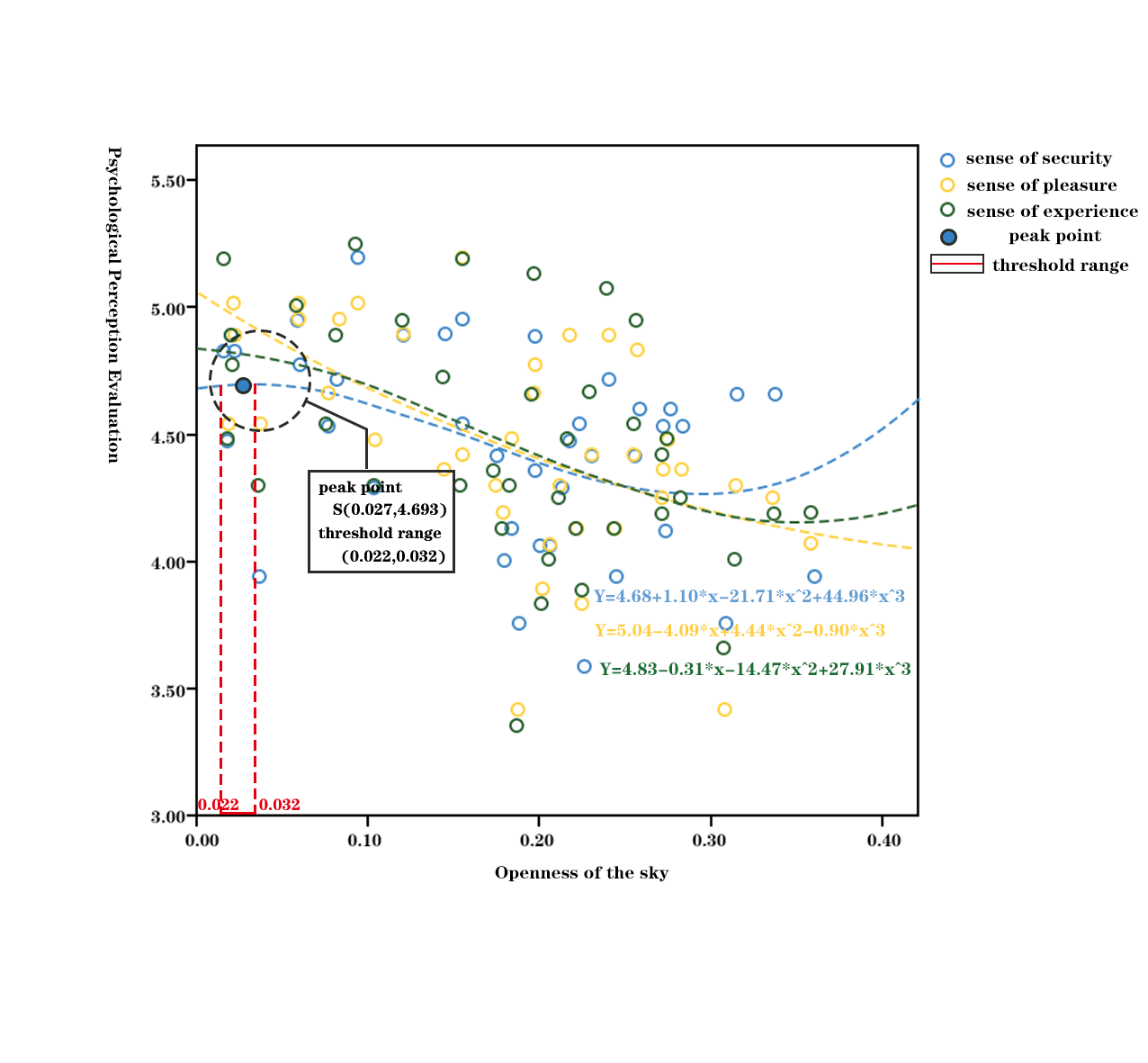


**Supplementary Figure 9.**Curve Fitting of the Openness of the Sky and Three Psychological Perceptions


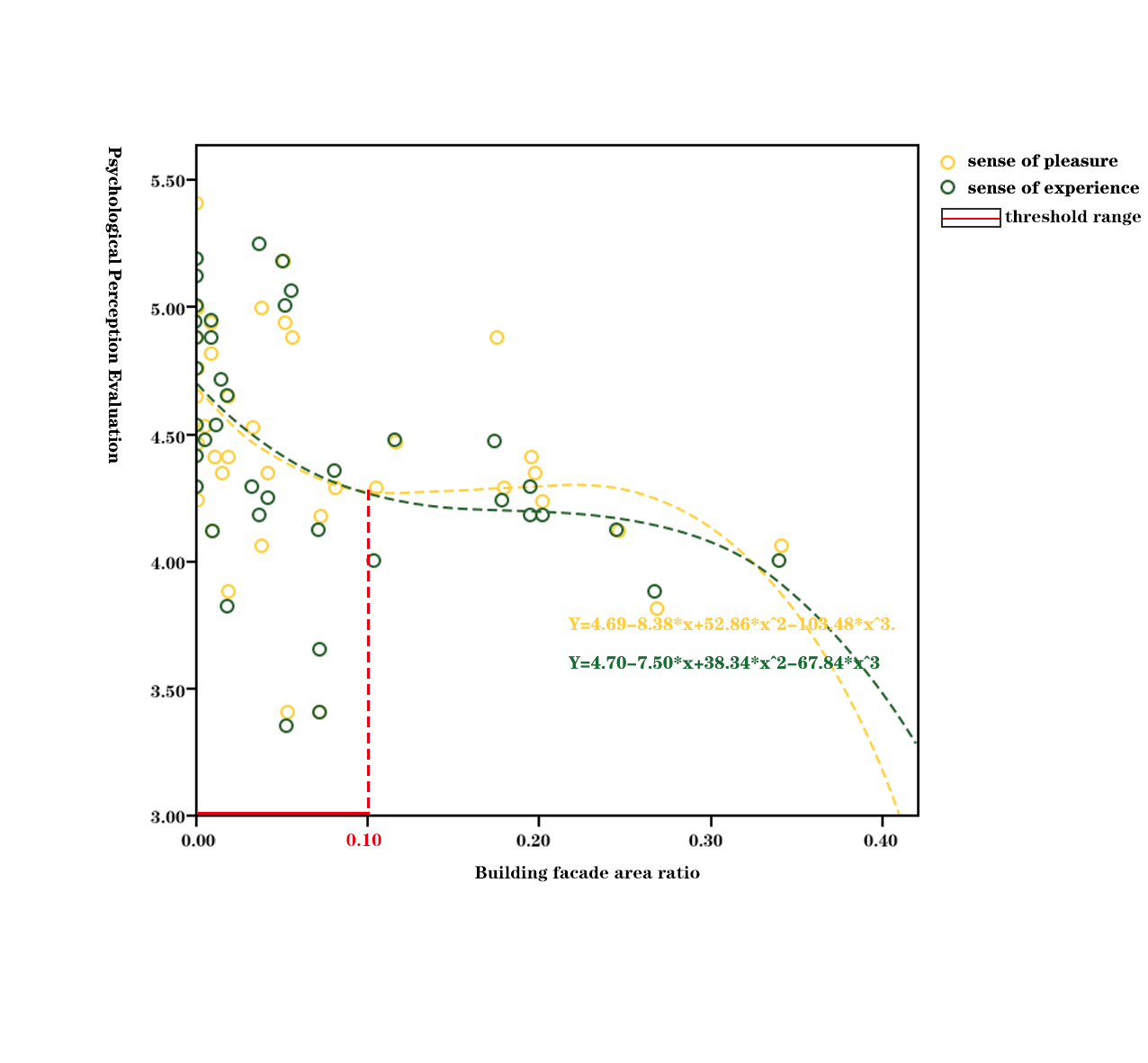


**Supplementary Figure 10.**Curve Fitting of the Building Facade Area Ratio and Two Psychological Perceptions


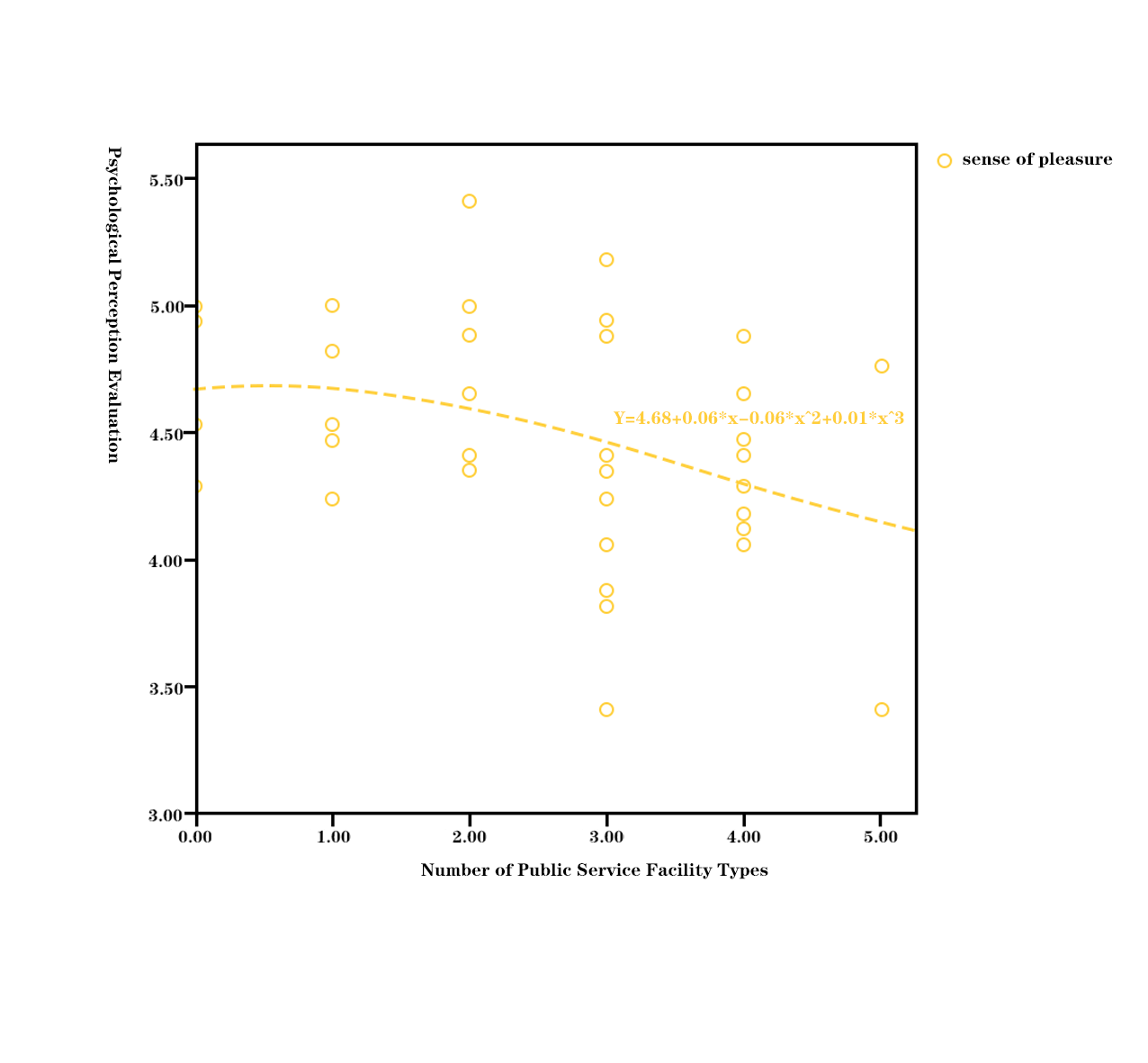


**Supplementary Figure 11.**Curve Fitting of the Number of Public Service Facility Types and Sense of Pleasure


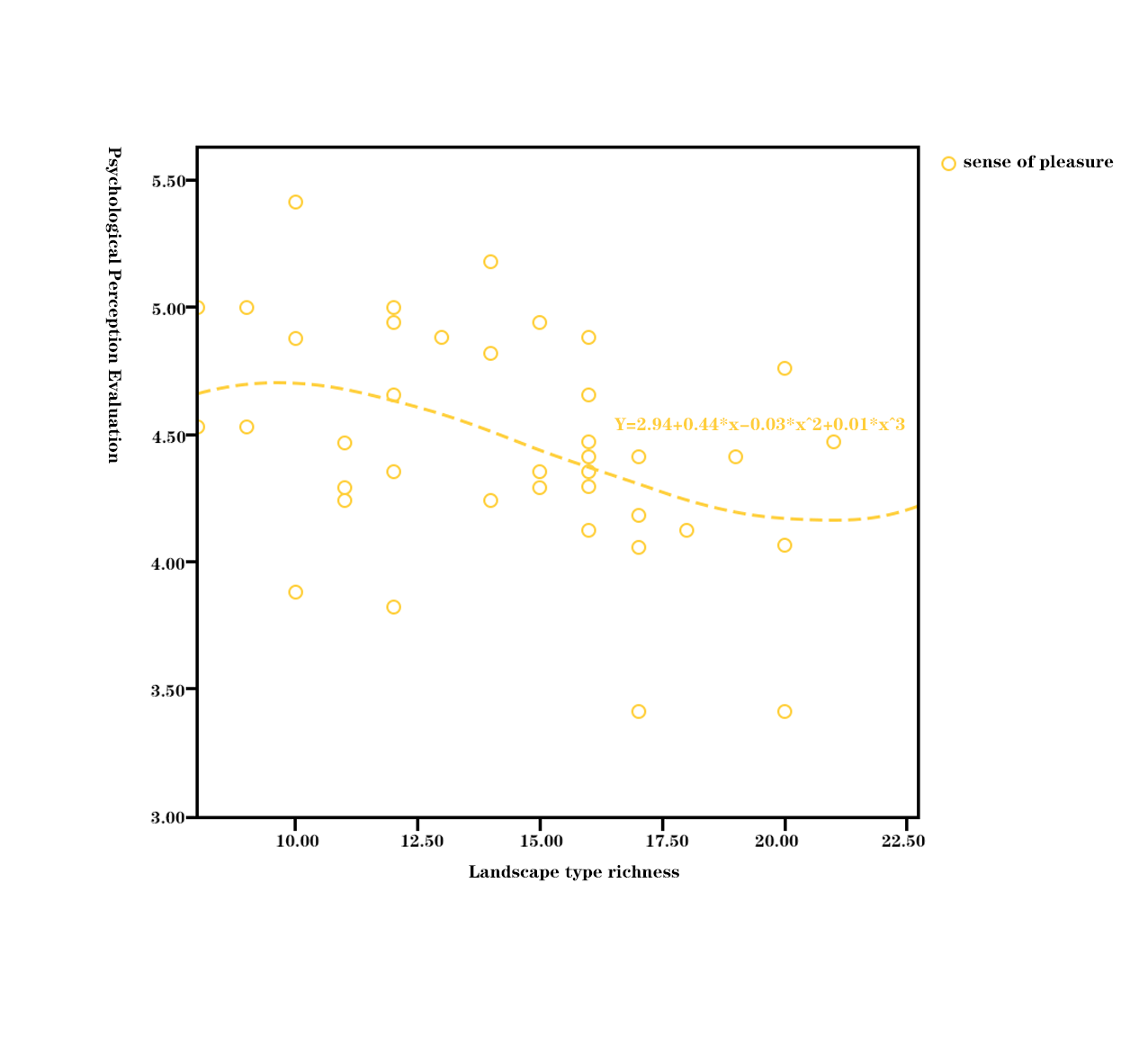


**Supplementary Figure 12.**Curve Fitting of Landscape Richness and Sense of Pleasure


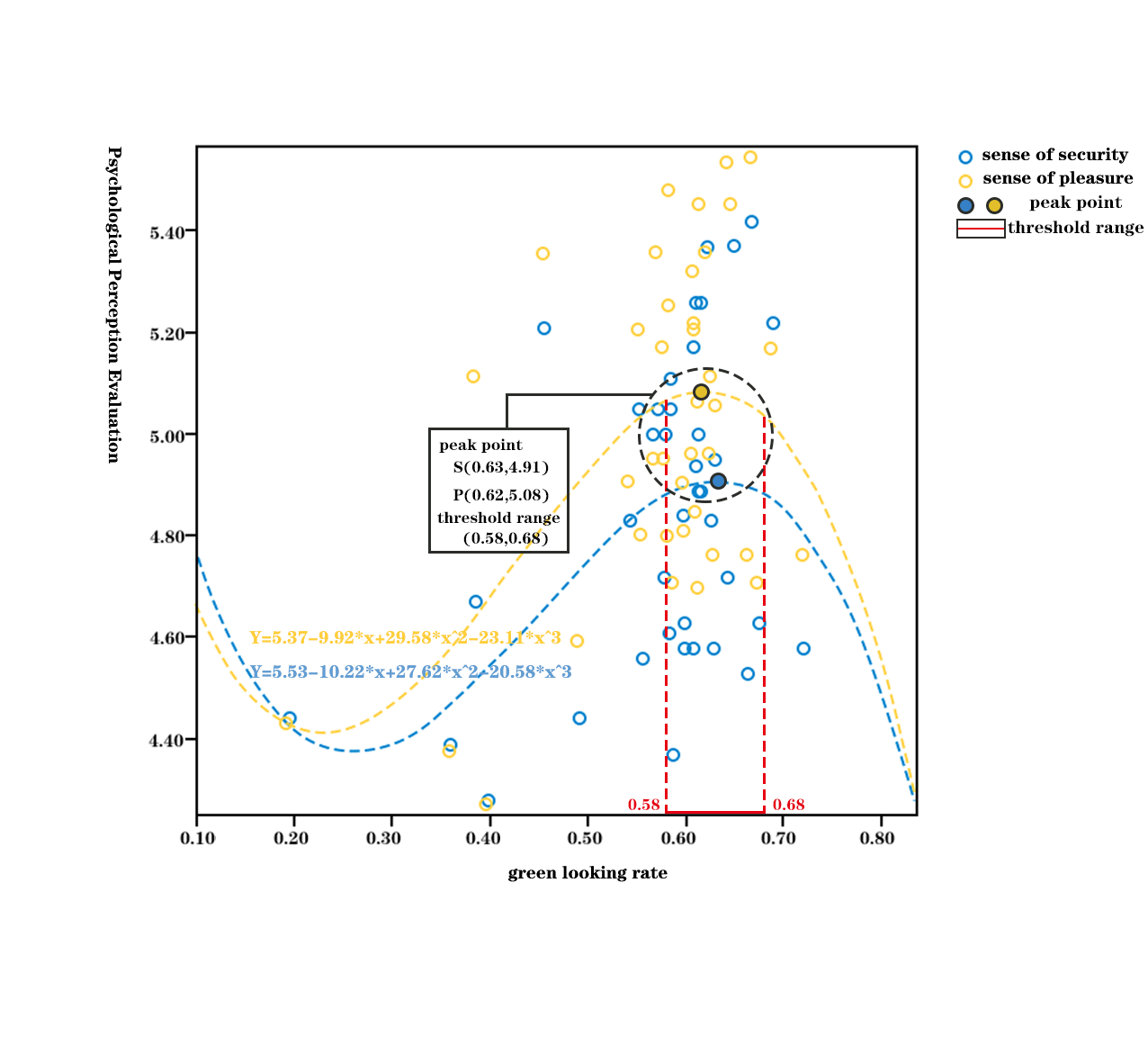


**Supplementary Figure 13.**Curve Fitting of the green-looking rate and Two Psychological Perceptions


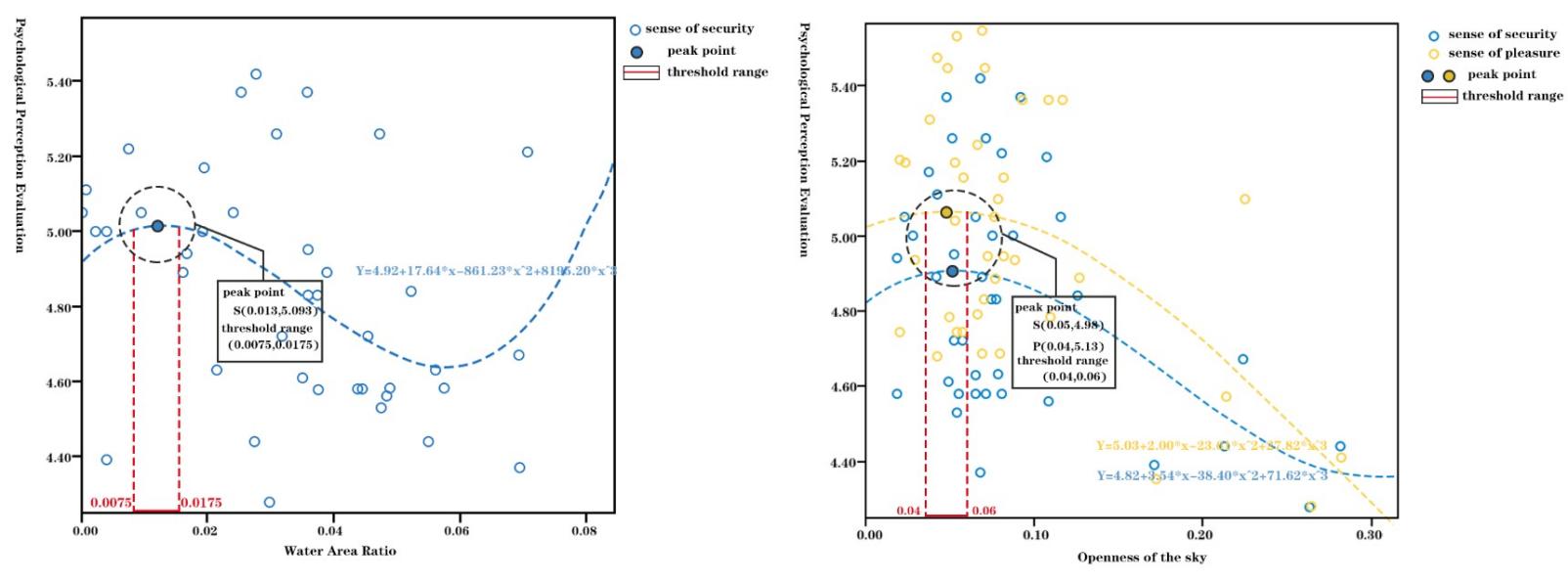


**Supplementary Figure 14.**Curve Fitting of the Water Area Ratio and Sense of Security and Curve Fitting of the Openness of the Sky and Two Psychological Perceptions


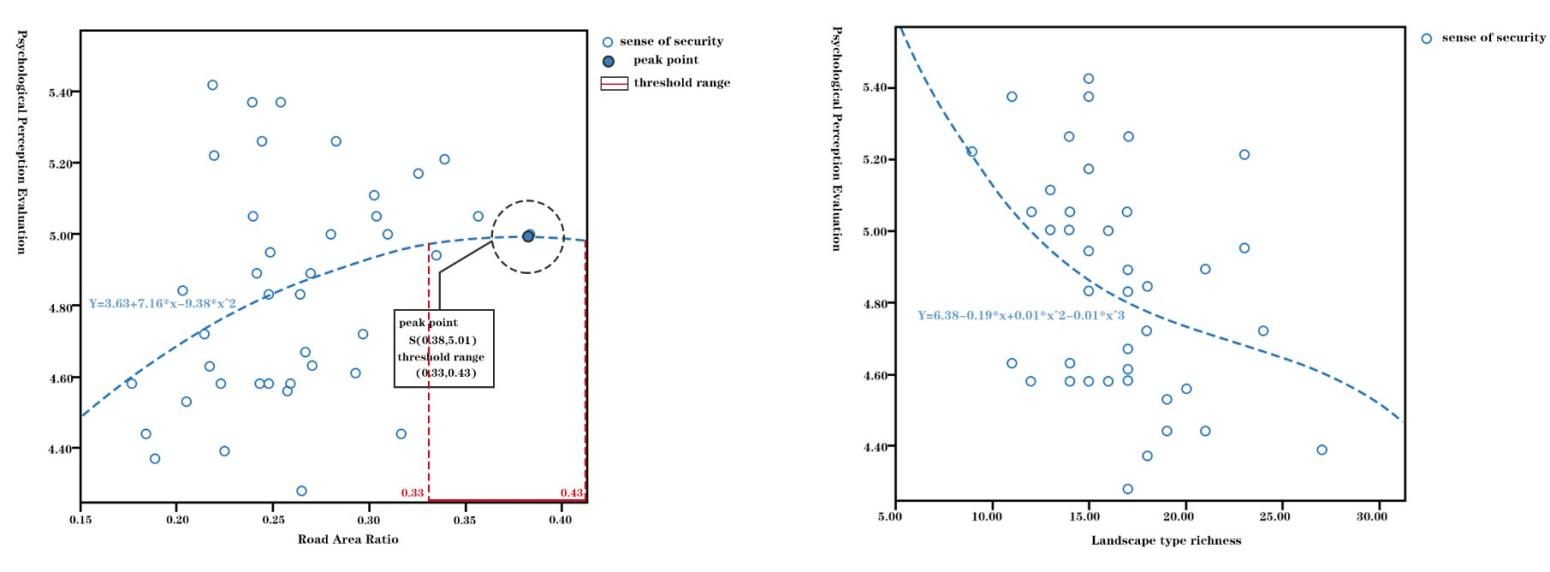


**Supplementary Figure 15.**Curve Fitting of the Road Area Ratio and Sense of Security and .Curve Fitting of Landscape Richness and Sense of Security

# Questionnaire mode

All the questionnaires in this study were designed and implemented in strict accordance with ethical standards. Before the research, we developed a detailed ethical process based on the relevant ethical norms. For all participants who completed the questionnaire or were interviewed, the research team fully informed them of key information such as research purpose, content, data usage, and privacy protection measures before the start, and provided written informed consent to ensure that participants fully understood and voluntarily participated in the study and had the right to withdraw from the study at any time, and their personal information would be strictly confidential, only used for the analysis of this study, and would not be leaked to any third party.

# 2.1Appendix A: Survey Questionnaire on Preferences for the Composition of Main Landscape Elements in Fitness Trails

Hello! This is a survey questionnaire on preferences for the composition of main landscape elements in fitness trails. The purpose is to gather your preferences regarding different landscape composition types along fitness trails. Please make your selections based on your intuitive feelings. This survey is for academic research purposes only, and your personal information will be kept strictly confidential. Please feel free to participate, and thank you for your involvement!

1.Gender:

A. Male B. Female

2. Age:

A. 6–20 B. 21–35 C. 36–60 D. Over 61

3. After observing the following real-life images and module combination diagrams, if you were to engage in activities within the fitness trail landscape, which type of space would you prefer? Please select:


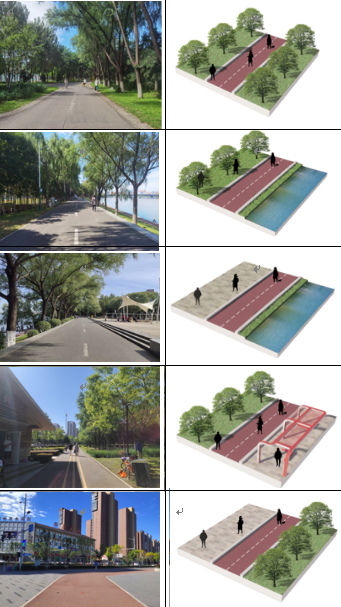
A.

B.

C.

D.

E.

1. Please briefly explain the reason for your choice:

# 2.2Appendix B: Semi-Structured Interview Outline on Visual Perception Characteristics of Fitness Trail Landscapes

**1.Purpose of the Interview:**

The aim of this semi-structured interview is to understand the visual perception characteristics of fitness trail landscapes from different user groups, with a focus on the elements that attract attention. The interview will help analyse people's preferences and needs regarding the landscape of fitness trails, providing valuable insights for the design and optimisation of fitness trail landscapes.

**2.Interview Method:**

The interview will be conducted in a semi-structured format, primarily using open-ended questions. Respondents will be encouraged to express their views freely, while the interviewer may guide the discussion as needed to explore deeper perceptions and insights.

**3.Interview Subjects:**

3.1. Users of fitness trails, including individuals of different ages, genders, and fitness levels.

3.2. Participants of various fitness activities (e.g., running, walking, cycling) and occasional users.

**4.Opening Statement:**

"Hello! Thank you for participating in our interview. The purpose of this interview is to understand your experiences and opinions regarding the landscape elements of fitness trails, particularly which elements capture your attention during your use of these spaces. Your feedback is very important in helping us evaluate the effectiveness of fitness trail landscape designs, and it will assist us in improving existing facilities to better meet user needs. During the interview, we will ask questions related to the landscape and environment, and we encourage you to answer based on your personal experiences."

**5.Specific Questions:**

I. Basic Information:

1. What is your age, gender, and your usual fitness habits?

2. When do you typically use fitness trails? (e.g., morning, afternoon, or evening)

II. Overall Impression of the Trail:

1. What was your first impression of the overall environment when you first walked into the fitness trail? What do you think is unique about the landscape design here?

2. While walking on the fitness trail, what elements catch your attention the most? Is it the natural landscape, man-made facilities, or something else?

III. Attention to Landscape Elements:

1. Do you pay particular attention to the surrounding landscape elements while using the trail? Which elements attract your attention the most? (e.g., greenery, trees, lawns, water features, sculptures, etc.)

2. Which visual elements (e.g., colours, materials, shapes) in the trail design enhance your experience? Please provide specific examples.

3. Do you choose a specific trail based on certain landscape elements? For example, if one trail has more greenery or better resting facilities, would you prefer to use that trail?

IV. Impact of the Landscape on Fitness Activities:

1. Do you think the landscape design of the trail affects your fitness activities? For instance, does it influence your running, walking, or cycling speed, or your mood?

2. In the landscape design, would you like to see more resting areas, open-view zones, or other functional facilities? Please share your thoughts.

V. Environmental and Psychological Perception:

1. When exercising on the trail, do the surrounding elements (such as greenery, landscape features, etc.) make you feel relaxed or energised? Which specific elements make you feel more relaxed or more energetic?

2. If the landscape design of the trail does not harmonise with the surrounding environment (e.g., nearby buildings, urban noise), would you feel uncomfortable? Does it affect your experience of the trail?

VI. Improvements and Suggestions:

1. What do you think are the shortcomings of the current fitness trail landscape? If you could suggest improvements, what areas would you focus on?

2. What landscape elements or facilities would you most like to see added in future trail designs?

**6.Closing Statement:**"Thank you very much for participating in our interview. Your opinions and suggestions are incredibly valuable to us. We will use your feedback to further improve the design of fitness trail landscapes, and we hope that future fitness trails will better meet your needs. Once again, thank you for your valuable time!"

Sure! Here's the translation of the provided text into British English:

# 2.3Appendix C: Fitness Activity Psychological Needs Survey Based on KANO Model

Assuming you will engage in activities such as walking or running on an outdoor fitness trail, please complete the following questionnaire regarding the feelings of pleasure, experience, comfort, sense of belonging, fun, safety, brightness, and warmth that the trail environment brings.

1.Gender:

A. Male B. Female

2. Age:

A. 6–20 B. 21–35 C. 36–60 D. Over 61

3.A sense of pleasure: A pleasant, relaxing, and enjoyable environment atmosphere.

If the surrounding environment brings you pleasure while engaging in activities, how would you rate it?

If the surrounding environment does not bring you pleasure while engaging in activities, how would you rate it?

A. Barely acceptable

B. Very enjoyable

C. Taken for granted

D. Indifferent

E. Very unpleasant

4.A sense of experienc: A convenient, reasonable, and pleasant environment atmosphere.

If the surrounding environment provides a good experience for your activities, how would you rate it?

If the surrounding environment does not provide a good experience for your activities, how would you rate it?

A. Barely acceptable

B. Very enjoyable

C. Taken for granted

D. Indifferent

E. Very unpleasant

5.A sense of comfort: A comfortable, smooth, and relaxing environment atmosphere.

If the surrounding environment brings you comfort, how would you rate it?

If the surrounding environment does not bring you comfort, how would you rate it?

A. Barely acceptable

B. Very enjoyable

C. Taken for granted

D. Indifferent

E. Very unpleasant

6.A sense of belonging: The feeling that the trail environment fosters a mutual connection with you.

If the surrounding environment brings you a sense of belonging, how would you rate it?

If the surrounding environment does not bring you a sense of belonging, how would you rate it?

A. Barely acceptable

B. Very enjoyable

C. Taken for granted

D. Indifferent

E. Very unpleasant

7.A sense of fun:An interesting, enjoyable, and novel environment atmosphere.

If the surrounding environment brings you a sense of fun, how would you rate it?

If the surrounding environment does not bring you a sense of fun, how would you rate it?

A. Barely acceptable

B. Very enjoyable

C. Taken for granted

D. Indifferent

E. Very unpleasant

8.A sense of security:A safe and stable environment atmosphere.

If the surrounding environment brings you a sense of safety, how would you rate it?

If the surrounding environment does not bring you a sense of safety, how would you rate it?

A. Barely acceptable

B. Very enjoyable

C. Taken for granted

D. Indifferent

E. Very unpleasant

9.A sense of brightness:A bright, tidy, and fresh environmental atmosphere.

If the surrounding environment brings you a sense of brightness, how would you rate it?

If the surrounding environment does not bring you a sense of brightness, how would you rate it?

A. Barely acceptable

B. Very enjoyable

C. Taken for granted

D. Indifferent

E. Very unpleasant

10.A sense of warmth:A warm, gentle, and cosy environmental atmosphere.

If the surrounding environment brings you a sense of warmth, how would you rate it?

If the surrounding environment does not bring you a sense of warmth, how would you rate it?

A. Barely acceptable

B. Very enjoyable

C. Taken for granted

D. Indifferent

E. Very unpleasant

# 2.4Appendix D: Psychological Perception Evaluation Questionnaire Based on the Semantic Differential (SD) Method (Sample Items)

We appreciate your valuable time and support for our research. Please respond to the questionnaire based on your genuine feelings. All data will be used solely for statistical analysis.

1. Gender:

A. Male B. Female

2.Age Group：

A. 6–20 B. 21–35 C. 36–60 D. Over 61

3 Please briefly review the following images to familiarise yourself with the general environment and overall layout of the experiment.


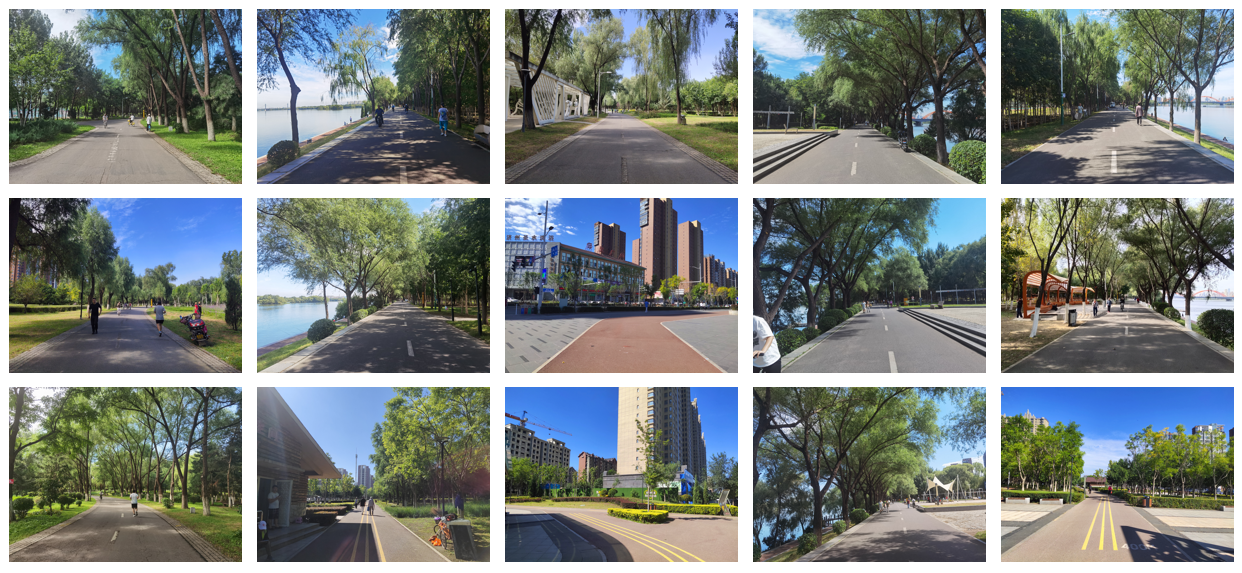


4. For each individual image, if you were to engage in activities such as walking or jogging in this environment, please evaluate the following three types of sensations.


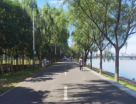


| sense of security is extremely strong | 7 | 6 | 5 | 4 | 3 | 2 | 1 | sense of security is extremely weak |
| --- | --- | --- | --- | --- | --- | --- | --- | --- |
| sense of pleasure is extremely strong | 7 | 6 | 5 | 4 | 3 | 2 | 1 | sense of pleasure is extremely weak |
| sense of experience is extremely strong | 7 | 6 | 5 | 4 | 3 | 2 | 1 | sense of experience is extremely weak |
